# Supplementary material for: Association of Serum BAFF Levels with Cardiovascular Events in ST-Segment Elevation Myocardial Infarction
Source: J Clin Med. 2023 Feb 20;12(4):1692. doi: 10.3390/jcm12041692 (PMC9964977; doi:10.3390/jcm12041692)
Supplement: Supplementary file 1 [file jcm-12-01692-s001.zip › jcm-2215811-supplementary.pdf]

Supplementary Materials

Association of Serum BAFF Levels with Cardiovascular Events in ST-Segment Elevation Myocardial Infarction

Running title: **BAFF Role in AMI Prognosis**

Title and page number of each element included in the document

Supplementary Tables	Page 2
Supplementary Figures	Page 4
Supplementary Methods	Page 7
Supplemental References	Page 9

Supplementary Tables

Table S1. Cox hazard models for Baff with MACEs in patients with STEMI

	Unadjusted HR (95%CI)	*P*-value	Adjusted for model 1 HR (95%CI)	*P*-value	Adjusted for model 2 HR (95%CI)	*P*-value
**Hospitalization for heart failure**						
**Log_2 BAFF**	1.635(1.000-2.671)	0.050	1.565(0.935-2.619)	0.088	1.403(0.813-2.422)	0.224
**BAFF cut-off**	3.180(1.377-7.345)	0.007	3.041(1.259-7.345)	0.013	2.581(0.977-6.819)	0.056
**Tertiles of BAFF**						
T1	1 (Ref)		1 (Ref)		1 (Ref)	
T2	1.886(0.552-6.444)	0.311	1.883(0.543-6.532)	0.319	1.829(0.529-6.322)	0.340
T3	3.4446(1.096-10.830)	0.034	3.248(0.964-10.944)	0.057	2.500(0.705-8.871)	0.156
**Nonfatal reinfarction**						
**Log_2 BAFF**	1.546(0.721-3.318)	0.263	1.623(0.727-3.624)	0.237	1.705(0.604-4.813)	0.313
**BAFF cut-off**	2.615(0.701-9.761)	0.153	2.866(0.713-11.524)	0.138	3.169(0.573-17.532)	0.186
**Tertiles of BAFF**						
T1	1 (Ref)		1 (Ref)		1 (Ref)	
T2	4.356(0.487-38.976)	0.188	4.899(0.536-44.751)	0.159	10.818(0.868-134.762)	0.064
T3	5.134(0.574-45.989)	0.144	6.208(0.628-61.361)	0.118	7.467(0.537-103.904)	0.135
**Stroke**						
**Log_2 BAFF**	1.521(0.804-2.879)	0.197	1.376(0.701-2.699)	0.353	1.190(0.605-2.339)	0.615

**BAFF cut-off**	2.751(0.923-8.194)	0.069	2.370(0.7511-7.477)	0.141	1.805(0.497-6.553)	0.370
**Tertiles of BAFF**						
T1	1 (Ref)		1 (Ref)		1 (Ref)	
T2	6.478(0.780-53.813)	0.084	6.018(0.711-50.939)	0.100	6.195(0.730-52.587)	0.095
T3	7.585(0.913-63.035)	0.061	6.502(0.731-57.817)	0.093	4.870(0.519-45.659)	0.166

BAFF were transformed into logarithmic form, a categorical variable using the lowest teritle as the reference.

Model 1 was adjusted for age and gender;

Model 2 was adjusted for model 1 and BMI, smoking, history of hypertension, history of DM, history of dyslipidemia, WBC, hsCRP and LVEF.

Abbreviation: BAFF, B-cell activating factor; BMI, body mass index; hs-CRP, high sensitivity C reactive protein; WBC, white blood cell; hs-CRP, high sensitivity C reactive protein; LVEF, left ventricular ejection fraction; DM, diabetes mellitus HR, hazard ratio; CI, confidence interval.

Supplementary Figures

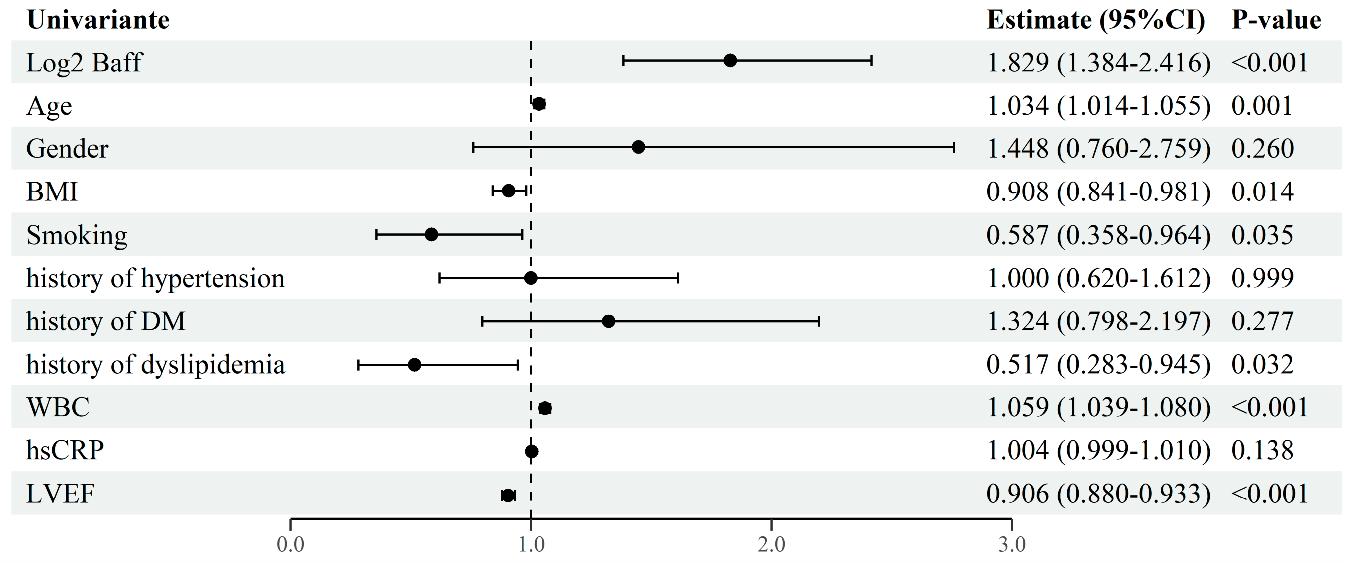

Figure S1. ***Univariate Cox analysis to examine the association between the BAFF levels and risk of MACEs.***

Abbreviation: MACEs, major adverse cardiovascular events; BAFF, B-cell activating factor; BMI, body mass index; hs-CRP, high sensitivity C reactive protein; WBC, white blood cell; LVEF, left ventricular ejection fraction; DM, diabetes mellitus HR, hazard ratio; CI, confidence interval.

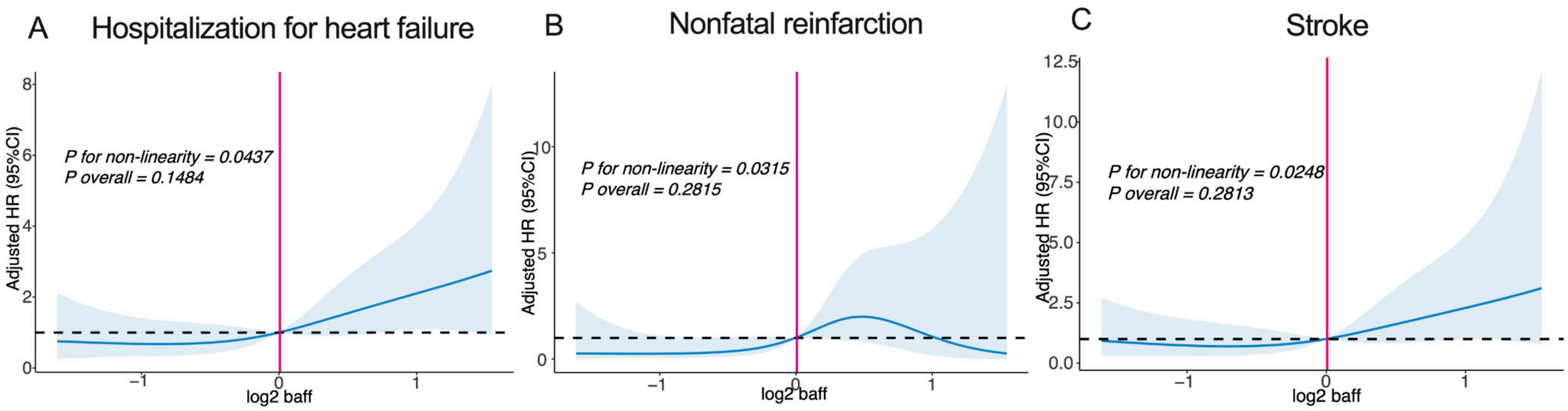

Figure S2. Dose-response relationship between BAFF levels and risk of the composite endpoint.

A, Dose-response relationship between BAFF levels and risk of hospitalization for heart failure; B, Dose-response relationship between BAFF levels and risk of nonfatal reinfarction; C, Dose-response relationship between BAFF levels and risk of stroke. Abbreviation: Restricted cubic spline curve was carried out with 4 knots at 5th, 25th, 75th and 95th percentiles of baseline BAFF levels. The reference point was the median of the BAFF in the 299 participants. The solid line represented point estimation on the association of BAFF with MACEs, and the shaded portion represented 95% CI estimation. Covariates in the model included age, sex, BMI, smoking, history of hypertension, history of DM, history of dyslipidemia, WBC, hs-CRP and LVEF. Abbreviation: MACEs, major adverse cardiovascular events; BAFF, B-cell activating factor; BMI, body mass index; hs-CRP, high sensitivity C reactive protein; WBC, white blood cell; LVEF, left ventricular ejection fraction; DM, diabetes mellitus HR, hazard ratio; CI, confidence interval.

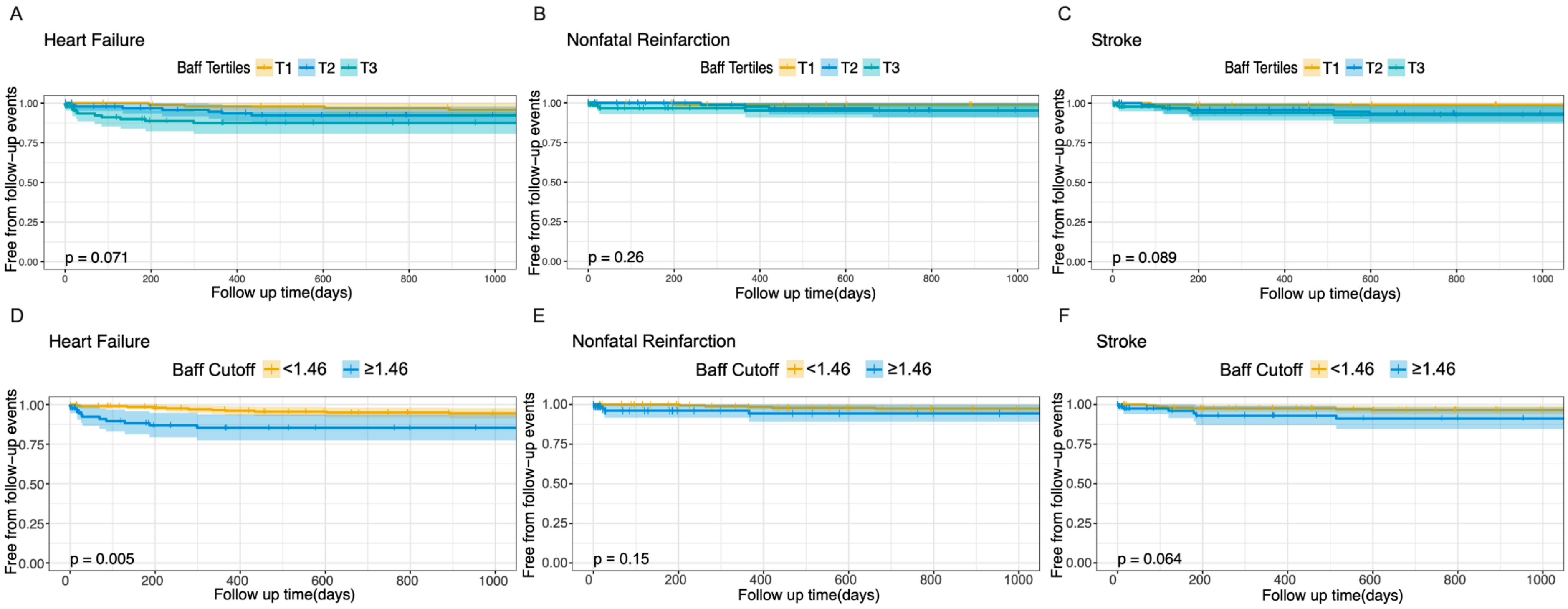

Figure S3. Kaplan-Meier curves of free from follow-up events stratifying by tertiles and cut-off of the BAFF levels.

A, Kaplan-Meier curve of free from follow-up hospitalization for heart failure stratifying by tertiles of the BAFF levels; B, Kaplan-Meier curve of free from follow-up nonfatal reinfarction stratifying by tertiles of the BAFF levels; C, Kaplan-Meier curve of free from follow-up stroke stratifying by tertiles of the BAFF levels; D, Kaplan-Meier curve of free from follow-up hospitalization for heart failure stratifying by cut-off value of the BAFF levels; E, Kaplan-Meier curve of free from follow-up nonfatal reinfarction stratifying by cut-off value of the BAFF levels; F, Kaplan-Meier curve of free from follow-up stroke stratifying by cut-off value of the BAFF levels. Abbreviation: MACEs, major adverse cardiovascular events; BAFF, B-cell activating factor.

Supplementary Methods

DEFINITION OF STUDY ENDPOINTS

Cardiovascular death

Death related to proximate cardiovascular causes (eg, myocardial infarction, cardiogenic shock, stroke, pulmonary embolism, ruptured aortic aneurysm, dissecting aneurysm or other causes), procedure-related complications, or any death unless an unequivocal non-cardiovascular cause could be established.

Hospitalization for heart failure

Hospitalization for heart failure (HF) is defined as an event where the patient is admitted to the hospital with a primary diagnosis of HF where the length of stay is at least 24 h, where the patient exhibits new or worsening symptoms of HF (dyspnea, decreased exercise tolerance, fatigue, worsened end-organ perfusion, or volume overload) on presentation, has objective evidence of new or worsening HF, and receives initiation or intensification of treatment specifically for HF.

Nonfatal reinfarction

Myocardial infarction is defined as spontaneous ST-segment elevation myocardial infarction or non-ST-segment elevation myocardial infarction according to the fourth universal definition.[1]

Any one of the following criteria meets the diagnosis of myocardial infarction:

- Detection of a rise and/or fall of cardiac biomarker values (preferably cardiac troponin) with at least one value above the 99th percentile URL and with at least one of the following:
    1. Symptoms of ischemia

2. (Presumed) new significant ST-T wave changes or new LBBB
3. Development of pathological Q waves
4. Imaging evidence of new loss of viable myocardium or new regional wall motion abnormality
5. Identification of an intracoronary thrombus by angiography or autopsy

- Cardiac death with symptoms suggestive of myocardial infarction and presumed new ischemic ECG changes or new LBBB, but death occurred before cardiac biomarkers were obtained, or before cardiac biomarker values would be increased.

Stroke

Stroke is defined as an acute symptomatic episode of neurological dysfunction, more than 24 hours in duration in the absence of therapeutic intervention or death, due to cerebral, spinal or retinal tissue injury as evidenced by neuroimaging or lumbar puncture. It includes the following subclassifications:

- Ischemic stroke
- Intracerebral hemorrhage
- Stroke of undetermined etiology

Supplemental Reference

1. Thygesen K, Alpert JS, Jaffe AS, Chaitman BR, Bax JJ, Morrow DA and White HD. Fourth Universal Definition of Myocardial Infarction (2018). *Circulation*. 2018;138:e618-e651.
